# Diversity of *Stomoxys* spp. (Diptera: Muscidae) and diurnal variations of activity of *Stomoxys indicus* and *S. Calcitrans* in a farm, in Wang Nam Khiao District, Nakhon ratchasima Province, Thailand

**DOI:** 10.1051/parasite/2012193259

**Published:** 2012-08-15

**Authors:** S. Keawrayup, G. Duvallet, S. Sukonthabhirom, T. Chareonviriyaphap

**Affiliations:** 1 Department of Entomology, Faculty of Agriculture, Kasetsart University Bangkok 10900 Thailand; 2 Centre d’Écologie fonctionnelle et évolutive (UMR 5175), Université de Montpellier France; 3 Office of Plant Protection Research and Development, Department of Agriculture, Ministry of Agriculture and Cooperative Bangkok 10900 Thailand

**Keywords:** *Stomoxys* spp., stable fly, diurnal variation of activity, Vavoua trap, Thailand, *Stomoxys* spp., mouche des étables, cycle d’activité diurne, piège Vavoua, Thaïlande

## Abstract

A study of species diversity of *Stomoxys* spp. and diurnal variations of activity of the most abundant was performed during a one year period at a local dairy cattle farm in Wang Nam Khiao District, Nakhon Ratchasima Province, Thailand. Four species of stomoxyine flies were morphologically identified, including *Stomoxys indicus* Picard 1908, *S. calcitrans* (Linnaeus 1758), *S. sitiens* Rondani 1873 and *S. uruma* Shinonaga and Kanao 1966. The most common species were *S. indicus* (50.2%) and *S. calcitrans* (49.5%). *S. sitiens* and *S. uruma* were found in small proportions (< 1%). The number of flies captured was significantly different among the three seasons with the greatest number in the rainy season (mean = 66%; df = 2, P < 0.05). The variations of diurnal activity were observed during different period of times (06:00 to 18:00) during three seasons. Both sexes of *S. indicus* and males of *S. calcitrans* showed unimodal activity pattern in cool and summer seasons. But a bimodal activity pattern was recorded in rainy season. For females *S. calcitrans*, a unimodal peak of activity was observed in cool season and a constant variation of activity all along the day in summer and rainy seasons, with an increase from the morning to the evening. A better understanding of stomoxyine fly behavior, especially the daily flight activity, can assist in prioritization and design of appropriate vector prevention and control strategies.

## Abstract (fr)

Une étude de la diversité des espèces de *Stomoxys* (Diptera : Muscidae) et des variations de l’activité diurne des deux espèces les plus abondantes a été menée pendant un an dans une ferme laitière du district de Wang Nam Khiao, Province de Nakhon Ratchasima, en Thaïlande. Quatre espèces ont été identifiées morphologiquement : *Stomoxys indicus* Picard 1908, *S. calcitrans* (Linnaeus 1758), *S. sitiens* Rondani 1873 et *S. uruma* Shinonaga et Kanao 1966. Les plus abondantes ont été *S. indicus* (50,2 %) et *S. calcitrans* (49,5 %). *S. sitiens* et *S. uruma* ont été trouvés en faible proportion (< 1 %). Le nombre de mouches capturées était significativement différent suivant les trois saisons, avec le nombre le plus important en saison des pluies (moyenne : 66 % ; ddl : 2 ; P < 0,05). Les variations diurnes du cycle d’activité ont été observées de 6 h 00 à 18 h 00 pendant les trois saisons. Les deux sexes de *S. indicus* et les mâles de *S. calcitrans* ont montré une activité unimodale en saison froide et en été, mais une activité bimodale en saison des pluies. Pour les femelles de *S. calcitrans*, une activité unimodale a été observée en saison froide, mais l’activité a été constante tout au long de la journée, avec une augmentation du matin au soir, en été et en saison des pluies. Une meilleure compréhension du comportement de ces mouches, spécialement leur cycle d’activité diurne, pourrait faciliter le développement de méthodes de prévention et de contrôle plus efficaces.

Stomoxyine flies are blood-sucking Diptera belonging to genus *Stomoxys* (Diptera: Muscidae), which contains eighteen different species in the world (Zumpt, 1973). One of these, *Stomoxys calcitrans* (Linnaeus 1758), normally referred as “stable fly”, is the most cosmopolitan species and a significant economic pest of livestock and other warm-blooded animals in many parts of the world (Zumpt, 1973; Greenberg, 1971; Harwood & James, 1979; Mullens *et al.*, 1988). Both male and female stable fly feed primarily on a wide vertebrate host range (Wall & Shearer, 1997). In the United Kingdom, *S. calcitrans* preferred to feed primarily on cattle and horses (Warnes & Finlayson, 1987). In Egypt, domestic donkeys and horses remain the most preferred vertebrate hosts (Hafez & Gamal-Eddin, 1959). Although livestock is a major blood source, humans can also be bitten by this species. When under mass attack of stable fly, significant economic losses due to reduction of anticipated gross weight gain and 30-40 % decrease in milk yields have been observed (Hall *et al.*, 1982; Mullens *et al.*, 1988). Campbell *et al.* (2001) reported weigh gains by grazing cattle were reduced an average of 0.20 kg per steer per day by an average of 2.79 flies per leg, representing a 19 % reduction in weight gain or 7 % per stable fly. In addition, stable flies have been known as mechanical vector for several pathogens such as *Anaplasma marginale* (anaplasmosis), *Trypanosoma* spp. (trypanosomosis) as well as different viruses, including bovine leucosis virus, lumpy skin disease virus (Mihok *et al.*, 1995; Torr *et al.*, 2006).

Surveys of adult stomoxyine fly populations can be assessed by different techniques. Various trapping devices have been developed to collect flies. In the United States, sticky traps (Broce trap and William trap) are commonly used for sampling stomoxyine flies (Williams, 1973; Broce, 1988). Recently, a friendlier field trap device, originally designed for tsetse fly collection in Africa, was used for stomoxyine fly collection. This “Vavoua trap”, whose name is derived from the name of an African village, has been proved to be a very efficient way to sample *Stomoxys* spp. in many African countries (Holloway & Phelps, 1991; Mihok *et al.*,1995; Mihok & Clausen, 1996), in La Réunion Island (Gilles, David, Duvallet, la Rocque and Tillard, 2007a) and in Thailand (Tainchum *et al.*, 2010, Muenworn *et al.*, 2010).

In Thailand, five species of stomoxyine flies are known (Zumpt, 1973; Masmeatathip *et al.*, 2006; Muenworn *et al.*, 2010). The presence of *S. pullus* is suspected, but not yet confirmed. *S. calcitrans* has been found in abundance in many parts of Thailand, particularly in the north and northeastern regions where dairy and beef cattle farms are most available (Sucharit & Tumrasvin, 1981). A good knowledge of biological and ecological data is absolutely crucial to understand the epidemiology of pathogen transmission by these flies and to design vector control methods. To assist in improving this base of information, we have studied the seasonal variations of density and daily variations of activity of stomoxyine flies in a local dairy cattle farm during a one year period of time.

## Materials and Methods

### Collection site

Stomoxyine flies collection was made at a local dairy cattle farm in Wang Nam Khiao District, Nakhon Ratchasima Province (14°25’6’’N, 101°51’0’’E). The majority of Wang Nam Khiao area is covered with organic farms near Tub Lan National Park, one of the biggest national parks in Thailand. Approximately 100 cows are housed in this local farm. Absolutely no insecticide has been used to protect cows from insect bites.

### Fly collection

Eight Vavoua traps (Laveissiere & Grebaut, 1990) were placed around the farm and left operational during the night before collection at 06:00 hr. Collections were made at every two hours at 06:00, 08:00, 10:00, 12:00, 14:00, 16:00 and 18:00 hr (local time) during two consecutive days per month from January to December in the year 2010. Captured flies were preserved in vials, containing 95 % ethanol and recorded by date and hour of capture. Specimens were subsequently brought back to the Department of Entomology, Faculty of Agriculture, Kasetsart University, Bangkok, Thailand for morphological identification following Zumpt (1973) with some modifications.

### Climatic parameters

Ambient air temperature and relative humidity were recorded every two hours at the dairy cattle farm, during the period of collections. And average rainfall was obtained at Nakhon Ratchasima meteorological station. Three seasons were categorized as: cool season (November to February), summer (March to June) and rainy season (July to October). Each season was of four months long and the same effort of flycollection (64 day-traps) was used per season.

### Data analysis

Captured flies were compared by a two-way analysis of variance (ANOVA). Differences among seasons on one side and day time periods on the other were performed, using Fisher’s least-significant difference. The accepted level of significance was determined at 5 % (P-value < 0.05). All data were analyzed using SPSS program package (Ver 17, SPSS Inc., Chicago, IL, USA).

## Results

A survey of stomoxyine flies was carried out at Wang Nam Khiao District, Nakhon Ratchasima Province, Thailand during a one year period from January to December 2010. Four species of stomoxyine flies were identified in this rural area, including *Stomoxys indicus* Picard 1908, *S. calcitrans* (Linnaeus, 1758), *S. sitiens* Rondani, 1873, and *S. uruma* Shinonaga & Kanao, 1966. A total of 3,449 flies were captured (Table I) with 1,731 specimens of *S. indicus*, representing 50.2 % of the total collection, and 1,707 specimens of *S. calcitrans*, representing 49.5 %. *S. sitiens* and *S. uruma* were found in a relatively low number with eight specimens (0.2 %) and three specimens (0.1 %), respectively.

**Table I.** Stomoxyine flies collected by Vavoua traps from a dairy cattle farm, Wang Nam Khiao District, Nakhon Ratchasima Province, Thailand (from January to December 2010).

	Number of flies		
Species	Male	Female	Total
*Stomoxys indicus*	556	1,175	1,731
*Stomoxys calcitrans*	1,193	514	1,707
*Stomoxys sitiens*	5	3	8
*Stomoxys uruma*	1	2	3

Total	1,755	1,694	3,449

The variations of seasonal abundance were determined during three different climatic seasons: cool, summer and rainy. In general, flies were found to be more abundant in the rainy period of the year (Table II). The total number of *S. indicus* and *S. calcitrans* captured in the rainy season was statistically different from other seasons (P < 0.05). A total of 75.5 % of *S. indicus* were captured in rainy season (1,307), followed by those captured in summer (221) representing 12.8 %, and in cool season (203) representing 11.7 %. Similarly, the greatest number of *S. calcitrans* were captured in rainy season (958) representing 56.1 % of the captures of this species. Lower proportions were collected in cool season (578) representing 33.9 % and in summer (171) representing 10 % (Table II). During all seasons, more females *S. indicus* were collected than males, while females *S. calcitrans* were collected in lower number compared to males as shown in Table II. The total number of *S. indicus* and *S. calcitrans* for both males and females were statistically different among the three seasons (P < 0.05). This analysis could not be made for *S. sitiens* and *S. uruma* because of the relatively low number of flies of these species captured for each season.

**Table II.** Total number of Stomoxyine flies (*Stomoxys* spp.) captured per season with Vavoua traps (64 day-traps per season) at Wang Nam Khiao District, Nakhon Ratchasima Province, Thailand.

	*S. indicus*		*S. calcitrans*		*S. sitiens*		*S. uruma*		
Season	Male	Female	Male	Female	Male	Female	Male	Female	Total
Cool^1^	74	129	386	192	2	0	1	2	786 (22.8%)
Summer^2^	79	142	90	81	2	0	0	0	394 (11.4%)
Rainy^3^	403	904	717	241	1	3	0	0	2,269 (65.8%)

1 Cool season: November-February

2 Summer season: March-June

3 Rainy season: July-October

**Fig. 1.**
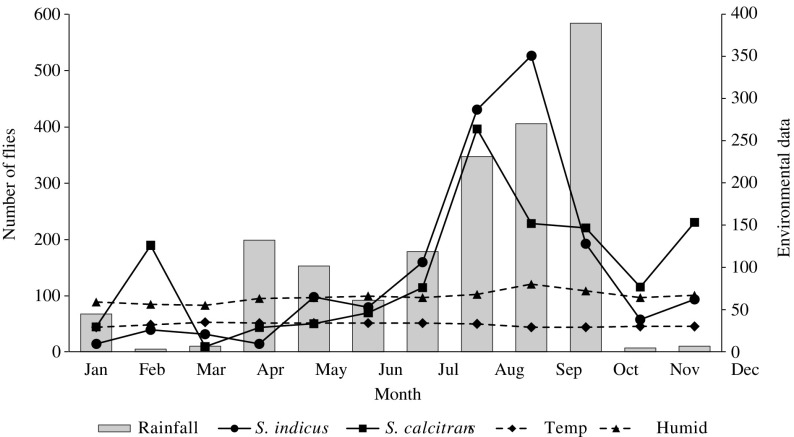
Monthly captures of *S. indicus* and *S. calcitrans* with Vavoua traps (16 day-traps per month) at Wang Nam Khiao District, Nakhon Ratchasima Province, Thailand (from January to December 2010), with indications of rainfall, temperature (Temp) and relative humidity (Humid).

Monthly fly collections were made during a one year period, and the total number of flies captured per species, per sex and per month was analyzed (Table III). The peak of abundance was observed in August for *S. calcitrans* (396 specimens: 329 males and 67 females), and in September for *S. indicus* (526 specimens: 127 males and 399 females) (Fig. 1). The lowest number of stomoxyine flies was collected in March in which 41 specimens of three species were captured. Three specimens of *S. uruma* were captured only in November. Significant differences in the total number of flies collected during the 12 months were observed (P < 0.05).

**Table III.** Monthly captures of Stomoxyine flies (*Stomoxys* spp.) with Vavoua traps (16 day-traps per month) at Wang Nam Khiao District, Nakhon Ratchasima Province, Thailand (from January to December 2010).

	*S. indicus*		*S. calcitrans*		*S. sitiens*		*S. uruma*		
Month	Male	Female	Male	Female	Male	Female	Male	Female	Total
January	7	7	32	12	2	0	0	0	60
February	14	25	129	60	0	0	0	0	228
March	8	23	3	6	1	0	0	0	41
April	7	7	20	23	1	0	0	0	58
May	31	66	26	24	0	0	0	0	147
June	33	46	41	28	0	0	0	0	148
July	51	108	75	39	0	0	0	0	273
August	137	293	329	67	0	2	0	0	828
September	127	399	154	74	0	1	0	0	755
October	88	104	159	61	0	1	0	0	413
November	17	40	69	46	0	0	1	2	175
December	36	57	156	74	0	0	0	0	323
Total	556	1,175	1,193	514	4	4	1	2	3,449

**Fig. 2.**
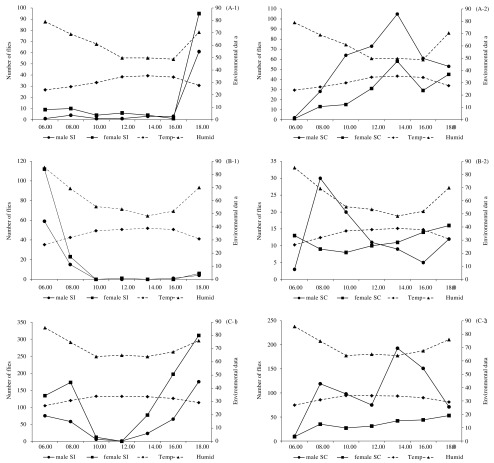
Variations of diurnal activity of *Stomoxys indicus* (SI) during cool (A-1), summer (B-1), rainy (C-1) seasons, and of *S. calcitrans* (SC) during cool (A-2), summer (B-2), rainy (C-2) seasons, collected with Vavoua traps (16 day-trap per month) at a Dairy Cattle Farm in Wang Nam Khiao District, Nakhon Ratchasima Province, Thailand.

Diurnal variations of activity of *S. indicus* and *S. calcitrans* during three seasons are given in Figure 2. Flies were collected from different periods of the day with two hours intervals, beginning from 06:00 to 18:00 hr. In cool season, a peak of activity was observed in evening (18:00 hr) for both males and females of *S. indicus* (Fig. 2, A-1). In contrast, a main peak of activity occurred in the afternoon (14:00 hr) for males and females of *S. calcitrans* (Fig. 2, A-2). For summer period, both sexes of *S. indicus* show a peak of activity in the morning at 06:00 hr (Fig. 2, B-1). Males of *S. calcitrans* showed a peak of activity in the morning (08:00 hr) but females of this species showed a constant activity all along the day with a slight increase in the afternoon (Fig. 2, B-2). During the rainy season, two peaks of activity were observed for males and females of *S. indicus*, with the most prominent before sunset (16:00 to 18:00 hr), and a less pronounced peak after sunrise (06:00 to 08:00 hr) (Fig. 2, C-1). Males of *S. calcitrans* showed also two peaks of activity with the most important in the afternoon (14:00 hr) and another one in the morning (08:00 hr), while females showed a constant activity throughout the day (Fig. 2, C-2).

## Discussion

Few studies of stomoxyine flies have been conducted in Southeast Asian countries, especially Thailand (Sucharit & Tumrasvin, 1981; Masmeatathip *et al.*, 2006; Muenworn *et al.*, 2010; Tainchum *et al.*, 2010). Most studies of stomoxyine flies have been well documented from Africa, the United States and France (Zumpt, 1973; Jones *et al.*, 1985; Gilles *et al.*,2007a, 2007b, 2008; Mavoungou *et al.*, 2008; Dsouli *et al.*, 2011a, 2011b). Recently, five species of stomoxyine flies were identified from Thailand in which the prevailing species in the rural setting area is *S. calcitrans* (Masmeatathip *et al.*, 2006; Muenworn *et al.*, 2010). In our study, four species of stomoxyine flies were identified, including *S. indicus*, *S. calcitrans*, *S. sitiens* and *S. uruma*.

*S. indicus* and *S. calcitrans* were found to be the most abundant in this local farm. *S. indicus* is considered as the most common *Stomoxys* species in the Oriental region (Zumpt, 1973). It has been recorded from many countries, from India and Sri Lanka in the West to Samoa Island in the East, and this species is commonly found in cattle barns (Zumpt, 1973). In Thailand, *S. indicus* was first reported by Masmeatathip *et al.* (2006) from Nakhon Pathom Province. In their work on the phylogeny of *Stomoxys* flies, Dsouli *et al.* (2011a) have shown that *S. indicus* could be the oldest species of *Stomoxys*, indicating that this genus could have originated in the Oriental region. In contrast, *S. calcitrans* is native to the Old World and known as a cosmopolitan species, commonly found in many areas in tropical and temperate zones. This species is regarded as a synanthropic fly, which followed human beings during their peregrinations everywhere in the world (Zumpt, 1973).

*S. sitiens* has been recorded from many places in the Ethiopian region ranging from the Gambia to Egypt all the way to South Africa, but this species is very rare in collections. It occurs also in the Oriental region from India to the Philippines, but the material is as rare as that from Africa (Zumpt, 1973). And *S. uruma* has been reported from the Iriomote and Ishigaki Islands, Ryukyus, Hong Kong and some specimens from India, Vietnam, Taiwan, and Thailand (Zumpt, 1973).

For the study of seasonal abundance, the results showed statistically different numbers (P < 0.05) between seasons. The greatest number of flies was captured during the rainy season while their number during the summer and cool seasons were not different. The high number of stomoxyine flies collected in this local dairy cattle farm is the consequence of appropriate environmental conditions, *i.e.* moisture, light intensity, rainfall, and temperature to maintain suitable breeding habitats. In the USA, a single seasonal peak of density for *S. calcitrans* was observed during the summer season, whereas marked bimodal and trimodal peaks have been documented in other locations (Mullens & Meyer, 1987; Lysyk, 1998). In Thailand, former observations showed that a peak of density of *S. calcitrans* was during the rainy season (Masmeatathip *et al.*, 2006; Muenworn et al., 2010). In our study, the greatest number of adult stomoxyine flies was captured during the rainy season as well. A major seasonal peak of abundance of *S. indicus* and *S. calcitrans* was found in this season. A minor peak has been observed in February, probably due to unusual important rainfalls in January 2010. The summer and cool seasons showed lower numbers of flies; that could be explained by the very low rainfalls and high temperature, which are unsuitable conditions for larval development (Zumpt, 1973). It should have been useful to extend such a survey on a 14-month period for a better explanation of the variations of fly density throughout the year. The differences observed in sex-ratios of our captures of *S. calcitrans* and *S. indicus* all along the year require further studies.

The variations of diurnal activity have been observed among different period of times (06:00 to 18:00) during three seasons. The patterns of activity between the most abundant species were quite different. Our results confirm the crepuscular activity of *S. indicus* already indicated by Zumpt (1973), who wrote that those flies are more active in the evening and they are readily collected by using light-traps set in cowsheds.

For *S. calcitrans*, the patterns during all seasons indicate a variation of diurnal activity. For males, this pattern is unimodal in cool season (peak in the afternoon) and summer season (peak in the morning), but bimodal in rainy season. For females *S. calcitrans*, a more or less constant activity was observed all along the day during all seasons. Many authors who have worked on the activity of stomoxyine flies focused only on *S. calcitrans*. Bimodal feeding activity patterns for *S. calcitrans* were reported by Mitzmain (1913), Simmonds (1944), Labrecque *et al.* (1975), Kunz & Monty (1976), and Charlwood & Lopes (1980). In contrast, Coaker & Passmore (1958) and Harley (1965) observed unimodal feeding activity patterns on daily feeding in Uganda. In Thailand, Masmethathip *et al.* (2006) reported that *S. indicus* showed the highest activity at sunset and dawn; in the same experiment, *S. calcitrans* showed an activity all through the day with a peak between 08:00 am to 10:00 am. Muenworn *et al.* (2010) observed a peak of flight activity of males *S. calcitrans* at 10:00 and 16:00 hr, whereas females showed an increase of activity all along the day until 16:00 hr. Berry & Campbell (1985) found that the pattern of daily activity of *S. calcitrans* was affected by temperature, humidity, and the level of solar radiation. In our study, *S. calcitrans* had the highest activity when temperatures range from 30 to 35 °C. This finding is the same as Hafez & Gamal-Eddin (1959), who worked on diurnal rhythm and seasonal variation, and reported that highest biting activity occurred about 30 °C.

## Conclusions

Our study confirmed that *S. indicus* and *S. calcitrans* are the most abundant species of *Stomoxys* in a local dairy local farm in Wang Nam Khiao District, Nakhon Ratchasima Province, Thailand. *S. indicus* appears in Asia as a vicariant species of *S. niger*, which is abundant in farms in Africa, along with the cosmopolitan *S. calcitrans*. It showed also that both species had their seasonal peak of abundance during the rainy season (August- September). And that their daily variations of activity was different during the seasons. Those results let us propose that, in this environment, control methods should be implemented in summer season to limit the development of their populations at the beginning of next rainy season. A better knowledge of larval breeding sites should help to control at the same time adult and larval stages.
